# Modular Synthesis of Bioreducible Gene Vectors through Polyaddition of *N*,*N*′-Dimethylcystamine and Diglycidyl Ethers

**DOI:** 10.3390/polym10060687

**Published:** 2018-06-20

**Authors:** Guoying Si, M. Rachèl Elzes, Johan F. J. Engbersen, Jos M. J. Paulusse

**Affiliations:** 1 Department of Biomolecular Nanotechnology, MESA+ Institute for Nanotechnology and TechMed Institute for Health and Biomedical Technologies, Faculty of Science and Technology, University of Twente, P.O. Box 217, 7500 AE Enschede, The Netherlands; guoyingsi@gmail.com (G.S.); M.R.Elzes@utwente.nl (M.R.E.); 2 20Med Therapeutics, Zuidhorst 251, Drienerlolaan 5, 7522 NB Enschede, The Netherlands; J.F.J.Engbersen@utwente.nl; 3 Department of Nuclear Medicine and Molecular Imaging, University Medical Center Groningen, P.O. Box 30.001, 9700 RB Groningen, The Netherlands

**Keywords:** cationic polymers, epoxy-amine reaction, bioreducible, gene delivery, disulfides, poly(amino ether)s

## Abstract

Bioreducible, cationic linear poly(amino ether)s (PAEs) were designed as promising gene vectors. These polymers were synthesized by the reaction of a disulfide-functional monomer, *N*,*N*′-dimethylcystamine (DMC), and several different diglycidyl ethers. The resulting PAEs displayed a substantial buffer capacity (up to 64%) in the endosomal acidification region of pH 7.4–5.1. The PAEs condense plasmid DNA into 80–200 nm sized polyplexes, and have surface charges ranging from +20 to +40 mV. The polyplexes readily release DNA upon exposure to reducing conditions (2.5 mM DTT) due to the cleavage of the disulfide groups that is present in the main chain of the polymers, as was demonstrated by agarose gel electrophoresis. Upon exposing COS-7 cells to polyplexes that were prepared at polymer/DNA *w*/*w* ratios below 48, cell viabilities between 80–100% were observed, even under serum-free conditions. These polyplexes show comparable or higher transfection efficiencies (up to 38%) compared to 25 kDa branched polyethylenimine (PEI) polyplexes (12% under serum-free conditions). Moreover, the PAE-based polyplexes yield transfection efficiencies as high as 32% in serum-containing medium, which makes these polymers interesting for gene delivery applications.

## 1. Introduction

Non-viral gene vectors are extensively sought after to deliver DNA as therapeutic agents to treat or cure genetic disorders [1,2,3,4,5,6,7]. The revolutionary advancement of CRISPR/Cas gene editing techniques has especially inspired renewed interest in the development of safe gene delivery agents [8,9,10]. Moreover, numerous non-viral vectors are able to deliver proteins which is a crucial step in the CRISPR/Cas process [11,12]. Several properties are required to render vectors attractive for gene delivery, such as high transfection efficiency, minimal or non-toxicity, and ease of manufacturing [13,14,15]. Cationic polymers are interesting candidates that are designed to meet these conditions, using modern developments in polymer chemistry [16,17,18,19] as well as nanotechnology [20,21,22]. These polymers spontaneously form polyplexes with DNA tightly packed inside by means of electrostatic interactions [23,24,25,26]. These interactions are aided by the release of sodium ions upon condensation through multivalent, polycationic polymers, leading to a net entropy increase [27,28,29]. After entering the cells through endocytosis, polyplexes must escape from the endosomal-lysosomal pathway and unpack the DNA in the cell nucleus in order to initiate gene expression [30,31,32,33]. The cationic nature of the polymers, which is often due to the incorporation of amine moieties, promotes endosomal escape by means of the proton sponge effect [34].

Many approaches were proposed to facilitate DNA unpacking, such as the use of acid-labile cationic polymers [35,36], ester-based cationic polymers [37,38], and biologically triggered cationic polymers [39,40,41,42]. Among these approaches, the latter approach is arguably the most interesting since it takes advantage of intracellular enzymes or related biomolecules [43,44]. Glutathione, an enzyme-related antioxidant that dictates the reductive potential of cells, is more concentrated inside the cells compared to the extracellular environment, which thereby increases the intracellular reductive potential [45,46,47]. Through the introduction of disulfide-linkages in cationic polymers, this higher reductive potential has been utilized as a trigger to cleave the disulfide bonds and unpack DNA from their polyplexes after the successful endosomal escape of the polyplexes into the cytosol [48,49,50].

The introduction of disulfide moieties was successfully employed in polyethylenimine (PEI) [51,52,53,54,55], poly(L-lysine) (PLL) [56,57], peptides [58,59], and poly(amido amine) dendrimers [60], resulting in increased gene transfection and improved biocompatibility. Green and coworkers reported cationic poly(amino ester)s with disulfides along the backbone for efficient gene delivery [61]. Our group, as well as other groups, reported on disulfide-linked linear and branched poly(amido amine)s that display excellent transfection efficiency and biocompatibility [62,63,64,65]. However, the clinical application of these polymers has so far been restrained due to their impaired transfection in the presence of serum [22,66]. The introduction of hydrophilic poly(ethylene glycol) (PEG) onto these vectors was found to increase the serum tolerance of these polymers to a limited extent, however they still failed to meet the requirements for clinical application [66,67]. Non-degradable poly(amino ether)s (PAEs), which are cationic polymers that bear ether groups that are similar to those of PEG, were shown to be effective gene delivery agents, however their serum tolerance was not investigated [68].

Here, we report a modular synthetic approach to generate disulfide-linked PAEs through the polyaddition of *N*,*N*′-dimethylcystamine (DMC) as a bifunctional diamine monomer, and through a series of diglycidyl ethers as epoxy monomers. The resulting bioreducible PAEs with structural varieties in the main polymer chain, will be evaluated for cell viabilities and transfection efficiencies.

## 2. Results and Discussion

### 2.1. Polymer Preparation and Characterization

**Scheme 1:**
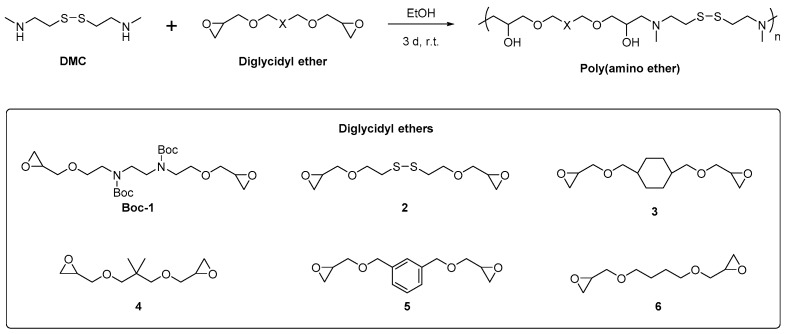
The modular synthesis of bioreducible poly(amino ether)s through the amine-epoxide reaction of *N*,*N*′-dimethylcystamine (DMC) with different diglycidyl ethers.

Cystamine is an interesting building block for biologically applied polymers, since its disulfide moiety renders the resulting polymers redox responsive. However, in combination with diglycidyl ethers, it may form branched or crosslinked structures due to the twofold reactivity of each of the primary amines [69,70]. The resulting structures suffer from poor water solubility, which impedes their application as gene vectors. In order to form linear polymers that are derived from cystamine disulfide, *N*,*N*′-dimethylcystamine disulfide (DMC), a cystamine analogue in which both amine groups are monomethylated was employed as the bifunctional amine monomer. The reaction of this amine with a series of diglycidyl ethers with structural variations yields water-soluble bioreducible linear poly(amino ether)s (PAEs) that are designed to be well applicable for efficient gene delivery (Scheme 1).

The set of diglycidyl ethers (DEs) consists of six different molecules, as depicted in Scheme 1. These are classified into three monomer groups: (i) high charge density (1); (ii) additional bioreducible linker (2); and (iii) varying hydrophobicity (3–6). All three factors are rationalized to play a role in the transfection performance of the cationic polymers. From these DEs, linear cationic PAEs were prepared by stirring the disulfide-containing diamine DMC with the corresponding diglycidyl ethers in the presence of excess triethylamine. The mixtures typically became homogenous after a couple of hours and subsequently became viscous, indicating the formation of polymers. No occurrence of gelation during the polymerization was observed, which indicated the formation of linear polymers. After removal of impurities and small oligomers through dialysis, the polymers were obtained in appreciable yields (ca. 14–45%). All of the polymer structures were validated by ^1^H NMR spectroscopy (see Figures S1–S6 in the supplementary information). No residual epoxide signals were observed in all cases. Molecular weights of the polymers were determined through size exclusion chromatography (SEC) and ranged from 2.5 kDa to 4.2 kDa, with polydispersities between 1.5–2.0 (summarized in Table 1).

**Figure 1:**
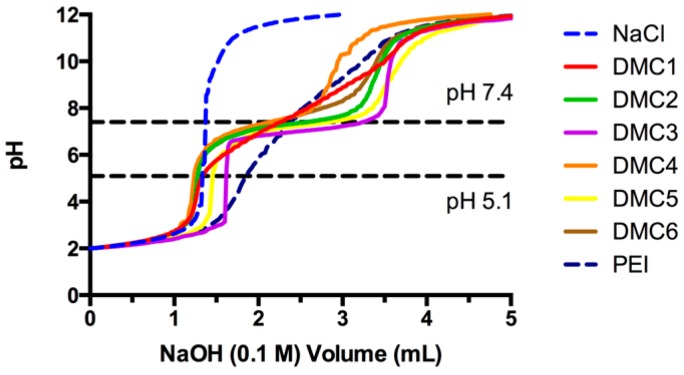
Titration curves of poly(amino ether)s by NaOH (0.1 M) from pH 2–12. PEI (branched, 25 kDa) and NaCl (150 mM) are included as references.

The obtained PAEs contain tertiary amines in their main chain that are prone to protonation in acidic milieu. Protonation not only grants the necessary positive charge to condense DNA into polyplexes, it also provides a buffer capacity to facilitate the endosomal escape of polyplexes through the proton sponge effect [34]. To evaluate the capacity of these polymers to accept protons in the endosomal acidification range (pH 7.4–5.1), titration experiments were performed. The titration curves for all PAEs are displayed in Figure 1, including the titration of reference component 25 kDa branched PEI, a commonly employed transfection agent, and blank NaCl solution (0.15 M). Plateaus along the titration curves in the pH 5.1–7.4 region are observed for all PAEs, which implies that there are pronounced buffer capacities. These buffer capacities range from 34% up to 64%, and are superior over PEI (18%). High buffer capacity of polymers is an excellent property to enhance endosomal escape which is necessary for the efficient gene delivery into the cytosol.

### 2.2. Polyplex Formation and Characterization

**Figure 2:**
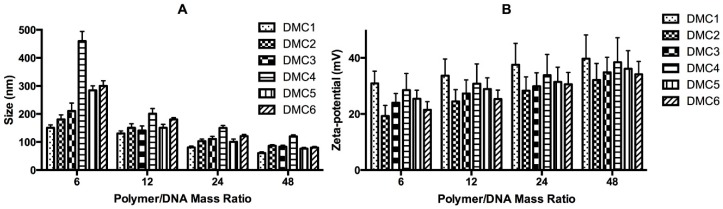
Characteristics of polyplexes prepared from bioreducible poly(amino ether)s and plasmid pCMV-GFP under various polymer/DNA mass ratios. (**A**) Hydrodynamic sizes versus mass ratios; (**B**) Zeta-potentials versus mass ratios.

For efficient transfection, naked DNA requires protection by vectors, which can be realized by forming polyplexes through electrostatic interaction between DNA and cationic polymers [71]. Here, polyplexes were prepared by combining polymer solutions of DMC1-6 and DNA solutions at polymer/DNA mass ratios of 6, 12, 24, and 48. The resulting size and surface charge of the obtained polyplexes at these ratios are displayed in Figure 2. The polyplexes that were formed at polymer/DNA ratios above 6 exhibit hydrodynamic sizes from 80 to 200 nm and zeta-potentials from +20 to +40 mV. With increasing polymer/DNA mass ratios, the polyplexes become smaller in dimension and carry increased positive charge, corresponding to more tightly packed polyplexes and an increased number of cationic PAEs incorporated in the formation of these polyplexes [23,26,72]. Polyplexes formed at a polymer/DNA mass ratio of 6 display a relatively large size (over 400 nm) and an average surface charge of +15 mV, which indicates a rather loose structure. Polyplexes prepared from DMC1 and DMC2 have a relatively small size compared to other polymers at the same polymer/DNA ratios. This phenomenon is related to the higher cationic density of polymer DMC1, which bears additional amine groups, resulting in increased electrostatic interaction with DNA. The additional disulfide content of polymer DMC2 makes this polymer more flexible along the main chain, since the disulfide bond has a low rotational barrier, which also leads to more compact polyplexes. The other four polymers DMC3, DMC4, DMC5, and DMC6 form polyplexes with similar values in size and zeta-potential at identical polymer/DNA mass ratios, and no clear relation can be drawn between the polymer structure and the characteristics of the polyplexes here.

**Figure 3:**
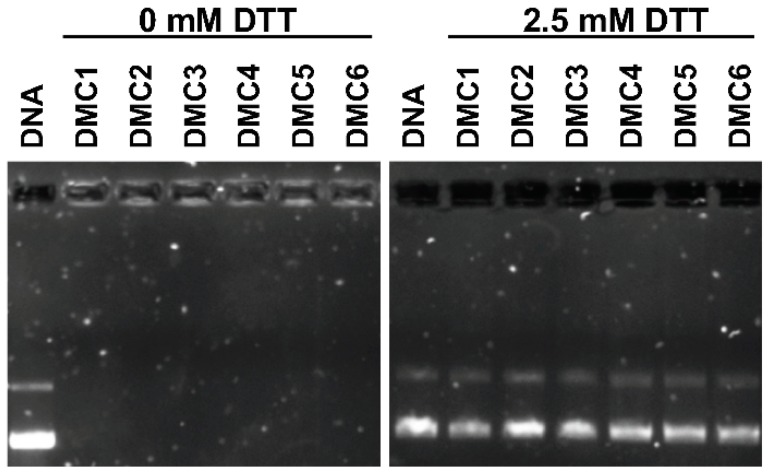
Agarose gel electrophoresis of polyplexes (*w*/*w* = 48) treated without DTT (**left**) and with 2.5 mM DTT (**right**).

The stability of the polyplexes under normal and reductive conditions was validated through agarose gel electrophoresis experiments, and results are shown in Figure 3. When untreated polyplexes (*w*/*w* = 48) that were dissolved in a HEPES buffer were subjected to electrophoresis, DNA bands did not migrate along the electric field, indicating that DNA was fully retained by the cationic PAEs. However, it is expected that due to the relatively high intracellular concentration of glutathione (GSH, 2.5–10 mM), the disulfides in the polymer chain can be cleaved resulting in the disassembly of the polyplexes and the release of the DNA content. This biological reaction was mimicked by the incubation of the polyplexes with dithiothreitol (DTT, 2.5 mM) as a reducing agent for 30 min under ambient conditions. These polyplexes displayed clear bands migrating along the electric force direction, similarly to free DNA, illustrating the release of DNA from the degraded polyplexes.

### 2.3. Cytotoxicity

**Figure 4:**
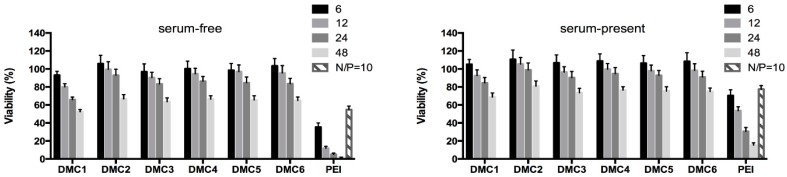
Cell viabilities of COS-7 cells exposed to polyplexes at different polymer/DNA mass ratios for 1h and incubated for 2 days, as determined via MTT assays under 0% serum conditions (**left**) and 10% serum conditions (**right**). Branched PEI (25 kDa) is included as a reference.

For biomedical applications, gene vectors require minimal toxicity and excellent biocompatibility towards cells and tissues [6,73]. It is therefore important to evaluate the cytotoxicity of the polyplexes before exploring their transfection performance. MTT assays were used to study the cytotoxicity profiles of the polyplexes towards COS-7 cells under serum-free (0%) and serum-present (10%) conditions. As shown in Figure 4, the viability of COS-7 cells under serum-free conditions is above 80% when treated with polyplexes prepared at polymer/DNA ratios of 6, 12, and 24 for all polymers, except for DMC1 which has the highest cationic density in the series due to the presence of extra secondary amino groups in the chain. In contrast, branched PEI (25 kDa) is considerably more toxic at the same polymer/DNA ratios. Under 10% serum conditions, cells show an even higher viability, and significant toxicity is only observed at the polymer/DNA mass ratio 48, indicating the promising potential of these PAEs for gene delivery under serum conditions.

### 2.4. Transfection Efficiency

**Figure 5:**
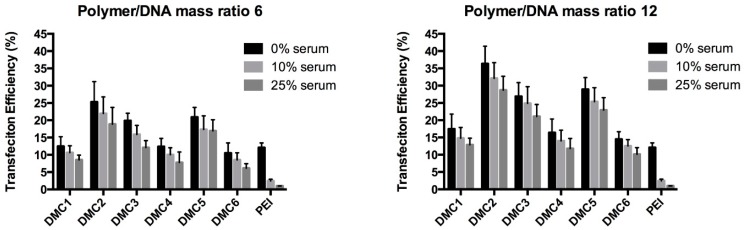
Transfection efficiencies of polyplexes prepared from bioreducible PAEs DMC1-6 and plasmid DNA at polymer/DNA mass ratios of 6 (**left**) and 12 (**right**). PEI is included as a reference at its optimal ratio of N/P = 10 in both graphs. COS-7 cells were exposed to polyplexes for 1 h and were incubated using various serum concentrations for 2 days. Transfection efficiency was quantified using FACS.

After the evaluation of the polyplex toxicity, the transfection performance of the polyplexes at polymer/DNA ratios 6 and 12 was investigated on COS-7 cells using green fluorescent protein (GFP) as a reporter gene. Three different serum conditions of 0%, 10%, and 25% were evaluated. In all experiments, PEI polyplexes at the same polymer/DNA ratios were included as a reference. Transfection efficiency was quantified by flow cytometry and was reported as the percentage of GFP-expressing cells of the total population (results are shown in Figure 5). Under serum-free conditions, the transfection efficiencies of the polyplexes prepared from PAEs at a polymer/DNA ratio of 6 were similar or enhanced (12–25%) compared to PEI (12.1%). The transfection efficiency increased at polymer/DNA ratio 12, reaching the highest efficiency of 36% for polyplexes based on DMC2. Relatively low transfection efficiencies were observed for DMC1, DMC4, and DMC6. This is most likely related to their lower buffer capacities as compared to polymers DMC2, DMC3, and DMC5. At polymer/DNA ratio 6, the transfection efficiencies of DMC2, DMC3, and DMC5 were 25 ± 5.9%, 20 ± 2.2%, and 21 ± 2.8%, respectively, which increased to 36 ± 5.1%, 27 ± 4.0%, and 29 ± 3.4%, respectively at a polymer/DNA ratio of 12.

As illustrated in Figure 5, the PAEs remained efficient in transfecting COS-7 cells, with efficiencies ranging from 10–32% at 10% serum and 10–28% at 25% serum, which is considerably better than that of PEI (2.5% at 10% serum and 1.0% at 25%). This effect of decreased transfection efficiency under serum-present conditions is consistently reported in literature, for example employing poly(amino ester)s [74], poly(amido amine)s [62], and several methacrylate-based cationic polymers [75]. The retained transfection of PAEs in the presence of serum is related to the hydroxyl groups that are present on the polymers causing a protein repelling effect, similar to polyethylene glycol [76]. A similar effect was reported for hydroxyl-functionalized methacrylates, where the addition of hydroxyls resulted in polyplex shielding against the serum, which was attributed to the formation of hydroxyl-DNA hydrogen bonding [77]. The transfection efficiency of DMC2-based polyplexes at a polymer/DNA mass ratio of 12 in the presence of 25% serum is 32%, and this is even further enhanced to 50% for polyplexes with a polymer/DNA mass ratio of 24 with a cell viability of up to 85% (data not shown). These results underline the high potential of these disulfide-based PAEs for non-viral gene delivery.

## 3. Materials and Methods

Epichlorohydrin (≥98%, Sigma-Aldrich, St. Louis, MO, USA), 2-hydroxyethyl disulfide (technical grade, Sigma-Aldrich), tetrabutylammonium bromide (TBAB, ≥98%, Sigma-Aldrich), sodium hydroxide (NaOH, pellets, Sigma-Aldrich), 1,3-benzenedimethanol (98%, Sigma-Aldrich), *N*,*N*′-dimethylethylene diamine (>97.0%, TCI Europe N.V., Zwijndrecht, Belgium), 1,4-butanediol diglycidyl ether (≥95%, Sigma-Aldrich), 1,4-cyclohexanedimethanol diglycidyl ether (**3**, Sigma-Aldrich), neopentyl glycol diglycidyl ether (**4**, Sigma-Aldrich), branched polyethylenimine (PEI, *M*_w_ 25 kDa, Sigma-Aldrich), dithiothreitol (DTT, Sigma-Aldrich), triethylamine (TEA, ≥99%, Sigma-Aldrich), borane dimethyl sulfide complex (BMS, Sigma-Aldrich), *N*,*N*′-bis(2-hydroxylethyl) ethylenediamine (>95.0%, TCI Europe N.V., Zwijndrecht, Belgium), di-*tert*-butyl dicarbonate (>95.0%, TCI Europe N.V.), and thiazolidine (98%, ACROS Organics) were used as received. The water used in these experiments was treated through a Milli-Q Gradient System (Millipore, Bedford, MA, USA). Plasmid DNA and plasmids pCMV-GFP and pCMV-ΔGFP, were purchased from a Plasmid Factory (Bielefeld, Germany).

^1^H NMR and ^13^C NMR were recorded on a Bruker NMR spectrometer (400 MHz) in deuterated solvents. The molecular weights were determined by size exclusion chromatography (SEC, Waters Alliance 2695) relative to PEG standards, using a Mixed-M column (PL-aquagel-OH 8 micron, 300 × 7.5 mm) with *N*,*N*-dimethylformamide containing LiCl (50 mM) as a mobile phase.

### 3.1. The Synthesis of N,N′-Dimethylcystamine (DMC)

The monomer was synthesized in its HCl salt form following our previous report [78]. Then, into a solution of thiazolidine (5.0 g, 56 mmol) in anhydrous tetrahydrofuran (THF, 20 mL) under a nitrogen atmosphere, borane dimethyl sulfide complex (BMS, 10.6 g, 140 mmol) in anhydrous THF (20 mL) was slowly added under stirring, and the mixture was subsequently refluxed overnight. After cooling down to the ambient temperature, methanol (20 mL) was added by syringe to neutralize any residual BMS, and the mixture was refluxed for an additional 3 h. After cooling down to the ambient temperature, the solution was purged with HCl gas, and the mixture was concentrated under reduced pressure. The resulting liquid was consecutively dissolved in methanol (5.0 mL) and was precipitated twice in diethyl ether (50 mL) to obtain a white oily precipitate. The precipitate was dissolved in water (7.5 mL) and was titrated with saturated I_2_/KI solution until a persistent yellow color was obtained. After stirring for an additional 2 h, the yellow solution turned colorless upon raising the pH to 10. The solution was extracted with chloroform (4 × 30 mL). Afterwards, the organic fractions were combined, dried over MgSO_4_, and were concentrated under reduced pressure to yield a clear liquid. Subsequently, the liquid was dissolved in isopropanol (20 mL) and was purged with HCl gas, yielding a turbid yellow mixture and a white precipitate. The precipitate was filtered off, washed with cold diethyl ether, and was finally recrystallized from isopropanol/methanol to obtain the HCl salt as a white solid. (2.12 g, 30.0% yield). ^1^H NMR (D_2_O, 400 MHz): δ_H_ 2.76 (s, 6H, CH_2_NHC**H**_3_), 3.05 (t, 4H, SC**H**_2_CH_2_), 3.44 (t, 4H, SCH_2_C**H**_2_). ^13^C NMR (D_2_O, 400 MHz): δ_C_ 31.92, 32.83, 47.02.

### 3.2. The Synthesis of N,N′-Bis(2-hydroxyethyl)-N,N′-bis(tert-butoxycarbonyl) ethylenediamine

Di-*tert*-butyl dicarbonate (17.6 g, 40 mmol) in dichloromethane (200 mL) was added dropwise under stirring at 0 °C to a mixture of *N*,*N*′-bis(2-hydroxylethyl) ethylenediamine (6.00 g, 40.0 mmol) and triethylamine (14.0 mL, 10.2 g, 100 mmol) in dichloromethane (100 mL). The mixture was stirred at the ambient temperature overnight. The solvent was removed under reduced pressure. The resulting solid was washed with ethyl acetate and diethyl ether and was subsequently dried under vacuum to yield a white solid (11.2 g, 80.4% yield). ^1^H NMR (CDCl_3_, 400 MHz): δ_H_ 1.45 (s, 18H), 3.25–3.60 (m, 8H), 3.71–3.82 (m, 4H).

### 3.3. The Synthesis of Diglycidyl Ethers

Diglycidyl ether monomers were synthesized via the reaction of epichlorohydrin with the corresponding diols. A typical example of the Boc-protected form of **1** (Boc-1) was: into a mixture of *N*,*N*′-bis(2-hydroxyethyl)-*N*,*N*′-bis(*tert*-butoxycarbonyl) ethylenediamine (1.75 g, 5.00 mmol), sodium hydroxide (0.800 g, 20.0 mmol), and tetrabutylammonium bromide (12.37 mg, 34.8 µmol), epichlorohydrin (1.45 mL, 18.4 mmol) was added gradually. The mixture was heated to 40 °C and was stirred for 3 h. The resulting mixture was subjected to column chromatography to obtain the diglycidyl ether of Boc-1 (1.95 g, 84.7% yield). ^1^H NMR (CDCl_3_, 400 MHz): δ_H_ 1.46 (s, 18H), 2.57 (s, 2H), 2.76 (t, 2H), 3.10 (m, 2H), 3.36 (m, 10H), 3.60 (m, 4H), 3.72 (m, 2H). ^13^C NMR (CDCl_3_, 400 MHz): δ 28.43, 44.12, 46.13, 47.34, 50.75, 69.89, 71.76, 79.76, 155.41.

Diglycidyl ether of **5** (1.12 g, 89.5 % yield). ^1^H NMR (CDCl_3_, 400 MHz): δ_H_ 2.62 (m, 2H, epoxide ring OC**H**HCHO), 2.81 (m, 2H, epoxide ring OCH**H**CHO), 3.20 (m, 2H, epoxide ring OCHHC**H**O), 3.45 (m, 2H, CHCH**H**O), 3.78 (m, 2H, CHC**H**HO), 4.59 (m, 4H, OC**H**_2_-phenyl ring), 7.27–7.36 (m, 4H, phenyl ring). ^13^C NMR (CDCl_3_, 400 MHz): δ_C_ 44.36, 50.93, 71.01, 73.27, 127.13, 128.64, 138.22.

### 3.4. The Synthesis of Poly(amino ether)s

Poly(amino ether)s (PAEs) were synthesized by the ring open polymerization of bifunctional epoxide monomers with equimolar amounts of primary amines. A typical procedure is given for the synthesis of DMC1:

Into a glass vial, diglycidyl ether Boc-1 (0.461 g, 1.00 mmol), DMC (0.254 g, 1.00 mmol), triethylamine (0.400 mL, 2.87 mmol), and ethanol (0.500 mL) were charged. After 3 d stirring under ambient temperature, the resulting viscous mixture was diluted with methanol (50.0 mL) and purged with HCl gas for 30 min in order to remove the Boc protecting groups. The mixture was concentrated, and the residue was dissolved in water (15.0 mL), acidified with 4 M HCl solution to pH 4, and was purified via dialysis (MWCO 1 kDa) against water overnight. Polymer DMC1 was obtained after lyophilization as an amorphous solid. The other PAE polymers DMC2-6 were synthesized analogously, however these polymers do not require the Boc removal step.

Polymer DMC1 (off-white solid, 0.162 g, 31.6%): ^1^H NMR (D_2_O, 400 MHz): δ_H_ 2.98 (s, 6H, NC**H**_3_), 3.10–3.16 (m, 4H, CH_2_NHC**H**_2_CH_2_O), 3.26 (m, 4H, NCH_2_C**H**_2_S), 3.32–3.38 (b, 8H, C**H**_2_NHCH_2_CH_2_O + OCH_2_CHOHC**H**_2_NCH_2_CH_2_S), 3.35–3.64 (m, 8H, C**H**_2_OC**H**_2_), 3.79 (t, 4H, OCH_2_CHOHCH_2_NC**H**_2_CH_2_S), 4.30 (bs, 2H, OCH_2_C**H**OHCH_2_NCH_2_CH_2_S).

Polymer DMC2 (white solid, 0.132 g, 33.2%): ^1^H NMR (D_2_O, 400 MHz): δ_H_ 2.95 (s, 6H, NC**H**_3_), 2.96 (m, 4H, OCH_2_C**H**_2_S), 3.09–3.14 (m, 4H, NCH_2_C**H**_2_S), 3.30 (m, 4H, CH_2_OC**H**_2_CHOHCH_2_NCH_2_CH_2_S), 3.55–3.65 (m, 8H, CH_2_OCH_2_CHOHC**H**_2_NC**H**_2_CH_2_S), 3.80–3.89 (m, 4H, OC**H**_2_CH_2_S), 4.25 (bs, 2H, CH_2_OCH_2_C**H**OHCH_2_NCH_2_CH_2_S).

Polymer of DMC3 (white solid, 0.086 g, 16.9% yield): ^1^H NMR (D_2_O, 400 MHz): δ_H_ 0.90–1.01 and 1.75–1.79 (m, 8H, 4 × C**H**_2_ of cyclohexane ring), 1.29 and 1.49 (m, 2H, C**H** of cyclohexane ring), 2.98 (s, 6H, NC**H**_3_), 3.11–3.18 (m, 4H, NCH_2_C**H**_2_S), 3.20–3.31 (m, 4H, CH_2_OC**H**_2_CHOHCH_2_NCH_2_CH_2_S), 3.37–3.47 (m, 4H, C**H**_2_OCH_2_CHOHCH_2_NCH_2_CH_2_S), 3.51–3.67 (m, 8H, CH_2_OCH_2_CHOHC**H**_2_NC**H**_2_CH_2_S), 4.25 (bs, 2H, CH_2_OCH_2_C**H**OHCH_2_NCH_2_CH_2_S).

Polymer DMC4 (amorphous solid, 0.064 g, 13.9% yield): ^1^H NMR (D_2_O, 400 MHz): δ_H_ 0.91 (s, 6H, C(CH_2_C**H**_3_)_2_), 2.92 (s, 6H, NC**H**_3_), 3.08–3.12 (m, 4H, NCH_2_C**H**_2_S), 3.26–3.32 (m, 8H, C(C**H**_2_CH_3_)_2_ + CH_2_OC**H**_2_CHOHCH_2_NCH_2_CH_2_S), 3.66–3.75 (m, 8H, CH_2_OCH_2_CHOHC**H**_2_NC**H**_2_CH_2_S), 4.25 (bs, 2H, CH_2_OCH_2_C**H**OHCH_2_NCH_2_CH_2_S).

Polymer of DMC5 (white solid, 0.220g, 43.7%): ^1^H NMR (D_2_O, 400 MHz): δ_H_ 2.96 (s, 6H, NC**H**_3_), 3.09–3.12 (m, 4H, NCH_2_C**H**_2_S), 3.24–3.30 (m, 4H, phenyl ring-CH_2_OC**H**_2_CHOHCH_2_NCH_2_CH_2_S), 3.57–3.64 (m, 8H, phenyl ring-CH_2_OCH_2_CHOHC**H**_2_NC**H**_2_CH_2_S), 4.26 (bs, 2H, phenyl ring-CH_2_OCH_2_C**H**OHCH_2_NCH_2_CH_2_S), 4.62–4.65 (m, 4H, phenyl ring-C**H**_2_OCH_2_CHOHCH_2_NCH_2_CH_2_S), 7.40–7.45 (m, 4H, phenyl ring).

Polymer of DMC6 (amorphous solid, 0.183 g, 40.1% yield): ^1^H NMR (D_2_O, 400 MHz): δ_H_ 1.63 (bs, 4H, OCH_2_C**H**_2_C**H**_2_CH_2_O), 2.96 (s, 6H, NC**H**_3_), 3.04–3.13 (m, 4H, NCH_2_C**H**_2_S), 3.23–3.30 (m, 4H, CH_2_OC**H**_2_CHOHCH_2_NCH_2_CH_2_S), 3.52–3.62 (m, 12H, C**H**_2_OCH_2_CHOHC**H**_2_NC**H**_2_CH_2_S), 4.25 (bs, 2H, CH_2_OCH_2_C**H**OHCH_2_N).

### 3.5. Buffer Capacity Titration

Buffering capacities of the polymers were determined via acid-base titration. The amount of PAE polymer equal to 5 mmol protonable amine groups was dissolved in NaCl aqueous solution (150 mM, 10 mL). The pH of the polymer solution was acidified to ≤2.0 and the solution was titrated with NaOH solution (0.1 M) using an automatic titrator (Metrohm 702 SM Tirino). As a reference, b-PEI (25 kDa) and NaCl aqueous solution were titrated following the same method. The percentage of amine groups protonated from pH 5.1 to 7.4 was defined as the buffer capacity that can be calculated from the following equation [79]:

(1) $$\mathrm{buffer}\;\mathrm{capacity}\;\left(\%\right)\;=\;\frac{\Delta V_{\mathrm{NaOH}}\;\times \;0.1\;M}{N\;\mathrm{mole}}\;\times \;100\%$$

wherein, ΔV_NaOH_ denotes the NaOH volume that is required to bring the pH value of the polymer solution from 5.1 to 7.4, and N mole (5 mmol) denotes the total moles of protonable amine groups in the PAE polymer.

### 3.6. Polyplex Preparation

Polyplexes were prepared by adding polymer solution into the DNA solution at designated polymer/DNA mass ratios. All polymer and DNA solutions were prepared in the HEPES buffer (20 mM, pH 7.4). The procedure of polyplexes prepared at the mass ratio of 48 is as follows:

In an Eppendorf tube (1.5 mL), DNA solution (75 µg/mL, 200 µL) and polymer solution (900 µg/mL, 800 µL) were successively added. The mixture was subjected to vortexing for 5 s and was incubated at room temperature for 30 min to obtain the polyplex suspensions. The size and surface charge of the polyplexes were measured on a Zetasizer Nano ZS (Malvern Instruments, Malvern, UK) at 25 °C. The data present the means of the three measurements with the corresponding standard deviations as error bars.

### 3.7. Agarose Gel Electrophoresis

Polyplexes were prepared at a polymer/DNA mass ratio of 48, as described in the previous protocol. Polyplex solutions (90 µL) were diluted with either DTT HEPES solution (25 mM, 10 µL) or HEPES buffer (10 µL), and were incubated under ambient conditions for 30 min. The resulting dispersions (20 µL) were mixed with 6× loading buffer containing bromophenol (Ferments, 5.0 µL) and were aliquoted (10 µL) to load on agarose gel (0.8% *w*/*v*) containing 1× SYBR^®^ Safe DNA Gel Stain (Invitrogen™, Carlsbad, CA, USA). The gel was developed at 90 V for 60 min in a TBE (Tris-borate-EDTA, 1×) running buffer and was consecutively imaged via FluorChem (Proteinsimple, Westburg, Leusden, The Netherlands) under UV excitation.

### 3.8. Cytotoxicity 

The cytotoxicity of polyplexes towards COS-7 cells was evaluated through MTT assays. In 96 well plates, ca. 10^4^ cells were seeded per well and were allowed to grow overnight in complete medium (100 µL, DMEM medium supplemented with 10% FBS) at 37 °C under 5% CO_2_ conditions to reach 60–80% confluency. The medium was replaced with fresh medium (100 µL, w/o 10% FBS). After 30 min of incubation, the cells were loaded with either polyplex solutions at various polymer/DNA mass ratios (100 µL, 0.25 µg DNA per well) or HEPES buffer (20 mM, pH 7.4, supplemented with 5.0 wt % glucose) and were incubated for an additional 1 h. All cells were refreshed with complete warm culture medium (100 µL) and were cultured for another 48 h. Half of the cells that were untreated with polyplexes were killed by incubating in 100× triton (4%, 10 µL) for 15 min, while the residual half were taken as a control to set the viability to 100%. All of the cells were washed with DPBS (100 µL) and were successively incubated in MTT solution (0.5 mg/mL, 100 µL) at 37 °C under 5% CO_2_ condition for 4 h. After refreshing MTT with DMSO (100 µL), the resultant formazone crystals were quantified through a plate reader (Tecan Infinite M200) at wavelength 540 nm with reference 680 nm. Measurements were performed in triplicate.

### 3.9. Transfection Efficiency

The plasmids of pCMV-GFP and pCMV-ΔGFP were respectively used as positive and negative controls. In 48 well plates, the cells were seeded at a density of 1.6 × 10^4^ per well (1.6 × 10^4^ cells/cm^2^) and were cultured in complete medium (DMEM with 10% FBS, 200 µL) at 37 °C under humidified atmosphere with 5% CO_2_ to reach confluencies of 60–80%. The cells were refreshed with warm medium (200 µL) with designated serum concentration and were maintained in new medium for 30 min until they were finally treated with polyplex solutions (200 µL, 0.5 µg DNA per well). After 60 min of incubation at 37 °C, the polyplexes were replaced with fresh warm complete medium (200 µL), and the cells were allowed to grow for another 48 h. After replacing the old medium with trypsin solution (0.25%, 200 µL), the cells were spun down (600 g, 5 min, R.T.), resuspended in HBSS buffer (200 µL), and were measured by the FACSCalibur (Becton-Dickinson, Breda, The Netherlands) at an excitation wavelength of 488 nm and emission at 530 nm. FACS Cellquest Software was applied to process the data. A 25 kDa branched PEI/DNA formulation, prepared at its recommended ratio of N/P = 10, was used as a reference.

## 4. Conclusions

A modular approach towards the incorporation of bioreducibility into linear poly(amino ether)s was developed through the reaction of *N*,*N*′-dimethylcystamine with various diglycidyl ethers. The obtained PAEs have good to excellent buffer capacities, ranging from 40 to 67%. Upon mixing PAEs with DNA at appropriate mass ratios, polyplexes were formed with hydrodynamic diameters ranging from 80–200 nm and zeta-potentials of up to +40 mV. The polyplexes displayed no significant toxicity towards COS-7 cells at polymer/DNA ratios up to 24 with transfection of COS-7 cells with notable efficiencies of up to 36%. In addition, the presence of serum did not markedly interfere with the transfection efficiency, even at serum concentrations of 10% and 25%. This effect is attributed to the presence of the protein repelling hydroxyl moieties within the PAEs. All transfection efficiencies and biocompatibility profiles of PAE-based polyplexes outpace the common polymeric vector PEI (branched, 25 kDa). Overall, these results present a novel modular approach towards a new generation of easily accessible cationic polymers for gene and drug delivery.

## Figures and Tables

**Table 1:** Characteristics of linear bioreducible poly(amino ether)s prepared through the reaction of *N*,*N*′-dimethylcystamine (DMC) with different diglycidyl ethers (1–6).

Poly(amino ether)	Yield ^a^ (%)	*M*_w_^b^ (kDa)	PDI ^b^	Degree of Polymerization ^b^	Buffer Capacity ^c^ (%)
DMC1	31.6	3.3	1.6	4.6	36.0
DMC2	33.2	4.0	2.0	4.4	50.4
DMC3	16.9	2.5	1.5	3.8	64.0
DMC4	13.9	2.9	1.7	4.1	34.4
DMC5	43.7	3.8	1.8	5.0	53.6
DMC6	40.1	4.2	1.8	6.1	36.8

^a^ Isolated polymer yield; ^b^ determined by size exclusion chromatography (SEC); ^c^ determined by titration, pH range 5.1–7.4.

## Supplementary Materials

The following supplementary materials are available online at http://www.mdpi.com/2073-4360/10/6/687/s1, Figures S1–S6: ^1^H NMR spectra of polymers DMC1-6.
