# Supplementary material for: Modular Synthesis of Bioreducible Gene Vectors through Polyaddition of N,N′-Dimethylcystamine and Diglycidyl Ethers
Source: Polymers (Basel). 2018 Jun 20;10(6):687. doi: 10.3390/polym10060687 (PMC6404356; doi:10.3390/polym10060687)
Supplement: Supplementary file 1 [file polymers-10-00687-s001.pdf]

Supplementary Information

Modular Synthesis of Bioreducible Gene Vectors Through Polyaddition of *N,N′*-Dimethylcystamine and Diglycidyl Ethers

Guoying Si,[1,†] *M. Rachèl Elzes,*[1,†] *Johan F.J. Engbersen*[2] *and Jos M.J. Paulusse*[1,3,*]

[1] Department of Biomolecular Nanotechnology, MESA+ Institute for Nanotechnology, Faculty of Science and Technology, University of Twente, P.O. Box 217, 7500 AE Enschede, The Netherlands

[2] 20Med Therapeutics, Zuidhorst 251, Drienerlolaan 5, 7522 NB Enschede, The Netherlands

[3] Department of Nuclear Medicine and Molecular Imaging, University Medical Center Groningen, P.O. Box 30.001, 9700 RB Groningen, The Netherlands

DMC1

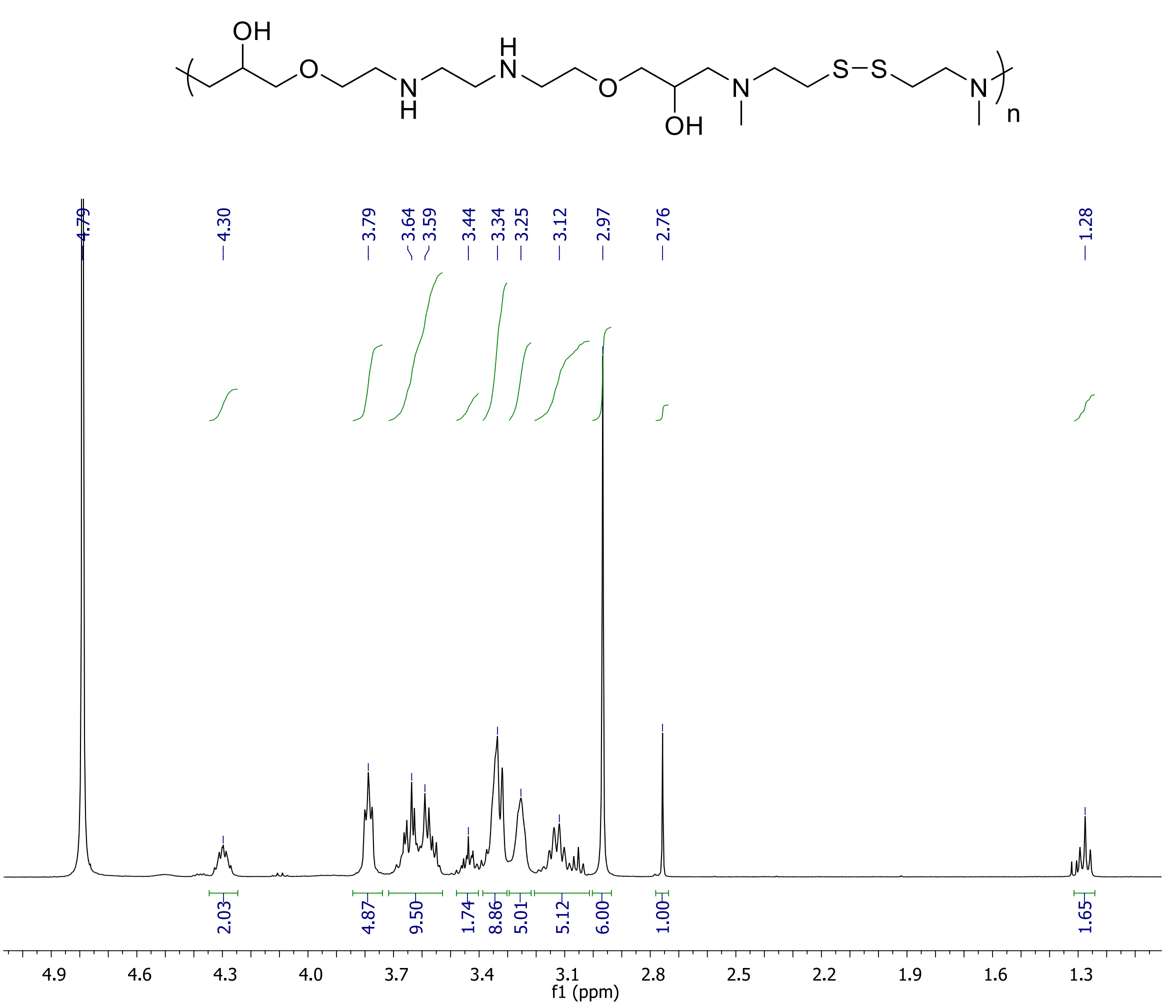

Figure S1. ^{1}H-NMR spectrum of DMC1 in D_2O.

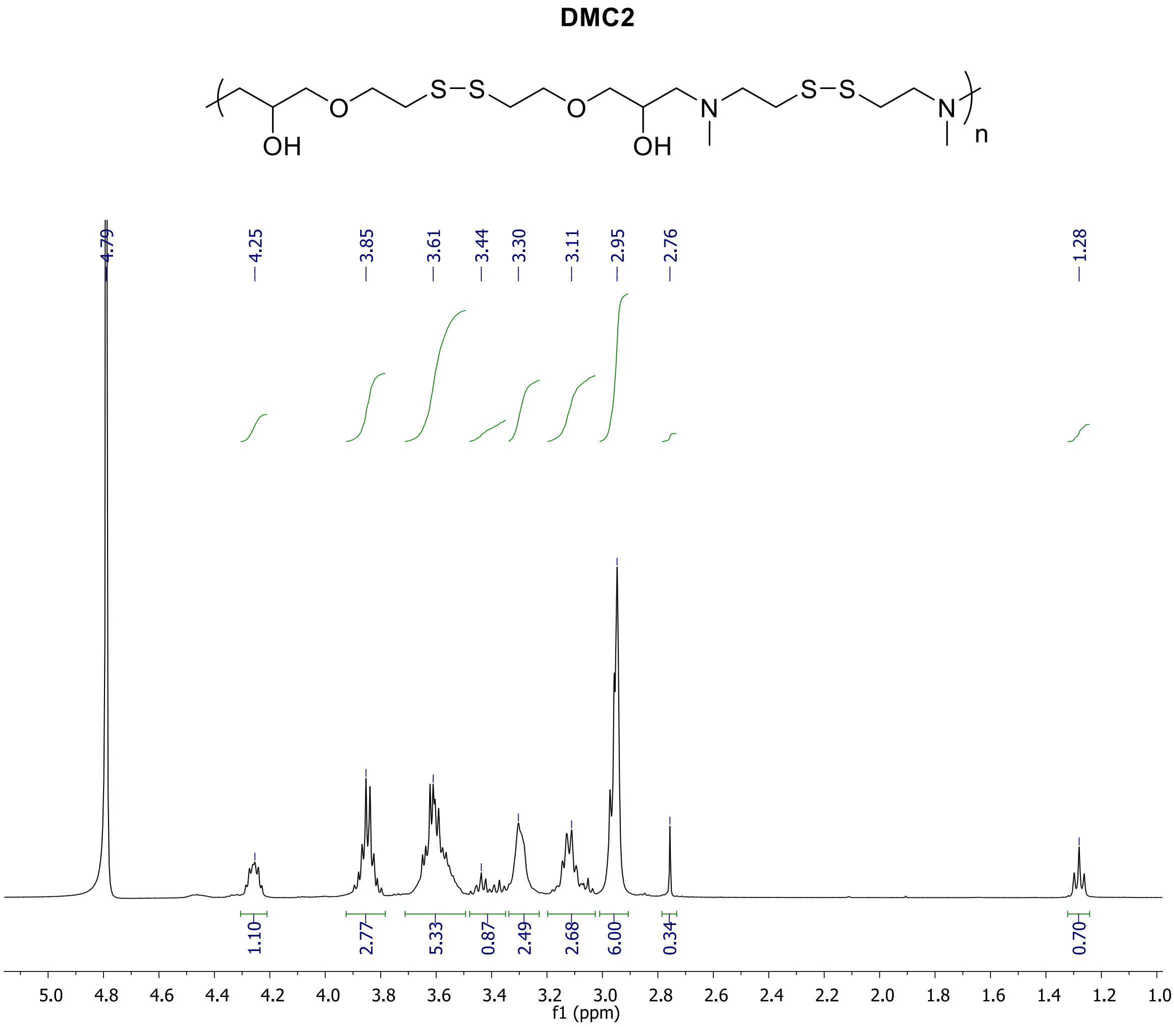

Figure S2. ^{1}H-NMR spectrum of DMC2 in D_2O.

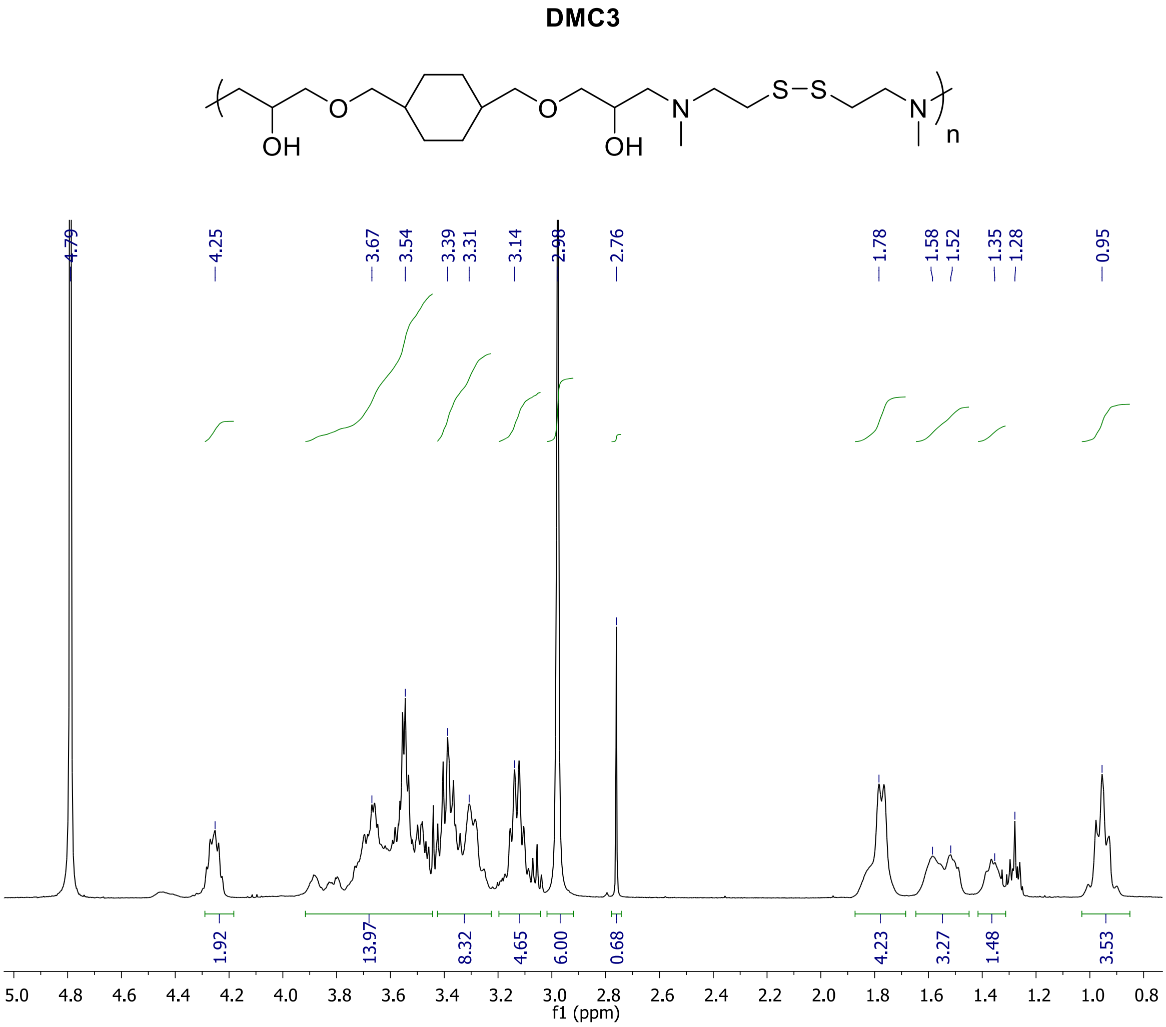

Figure S3. ^{1}H-NMR spectrum of DMC3 in D_2O.

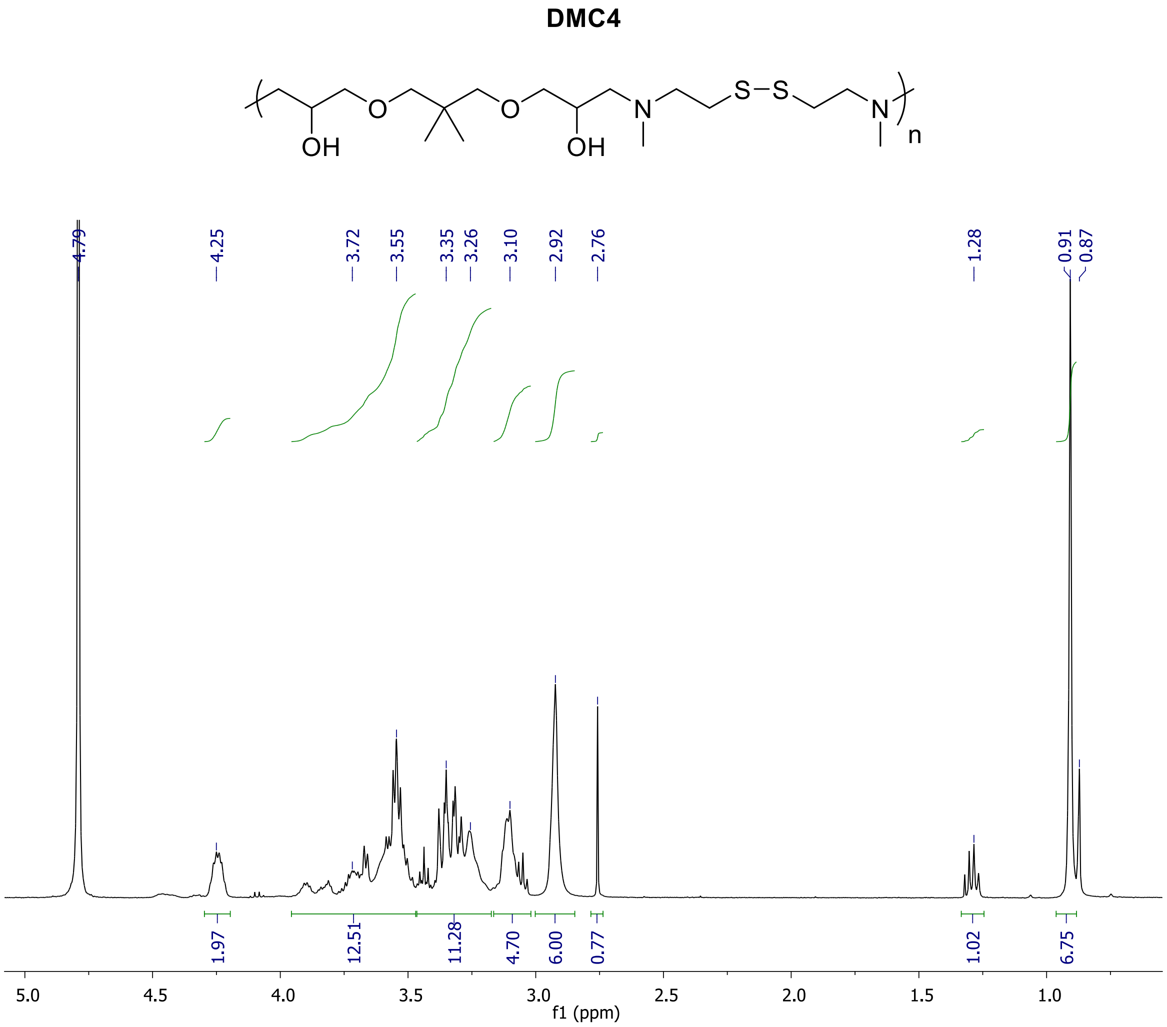

Figure S4. ^{1}H-NMR spectrum of DMC4 in D_2O.

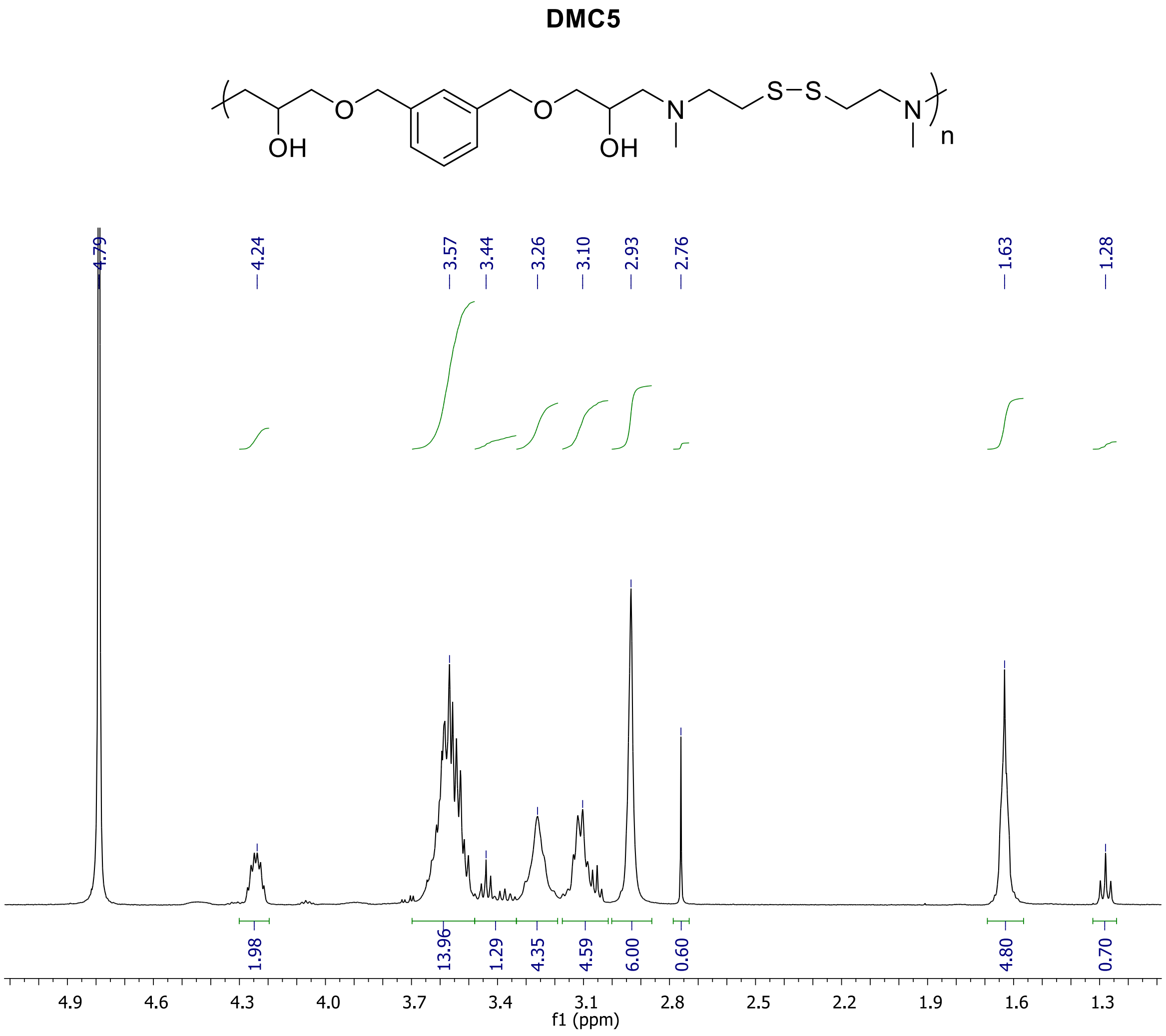

Figure S5. ^{1}H-NMR spectrum of DMC5 in D_2O.

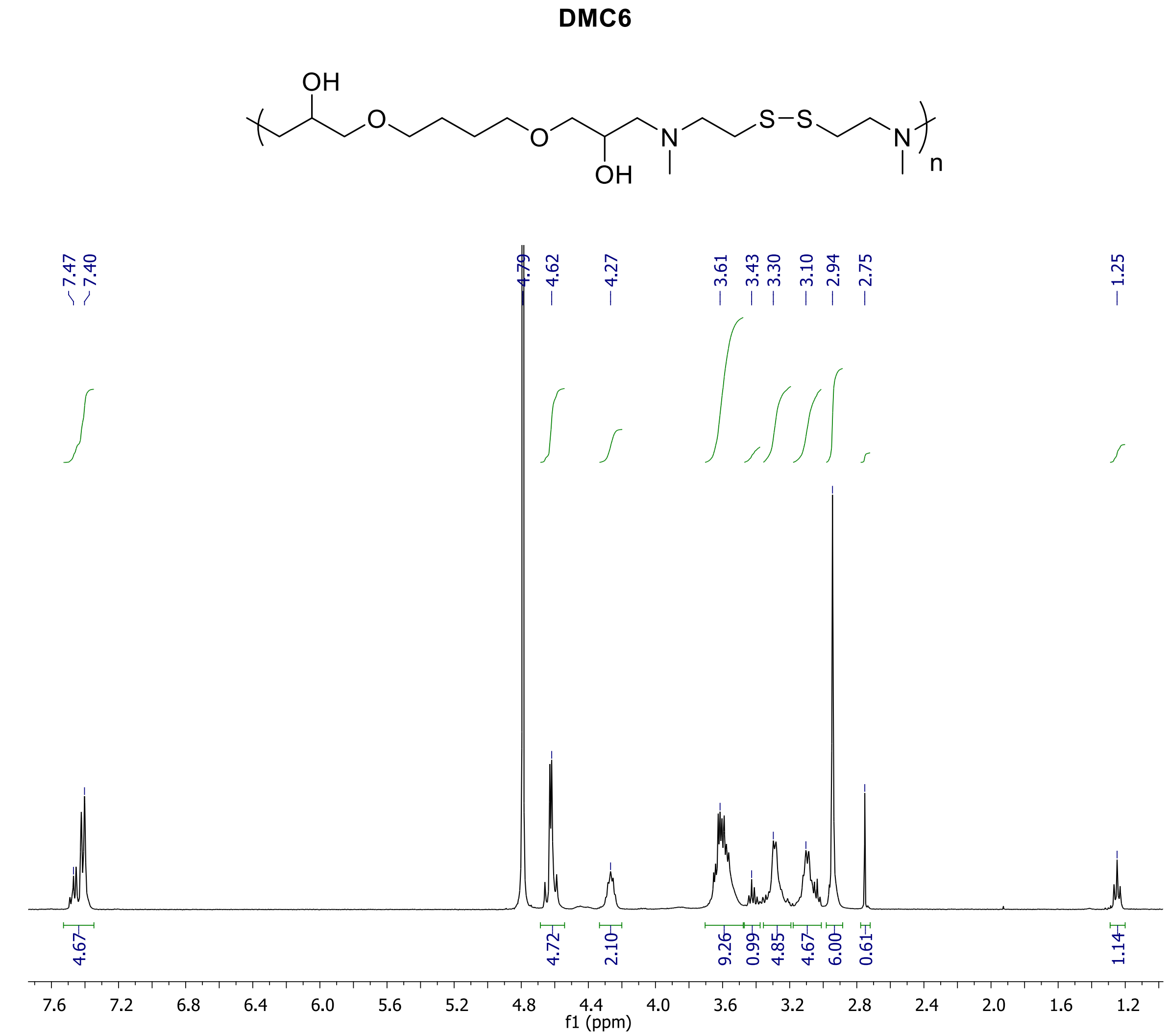

Figure S6. ^{1}H-NMR spectrum of DMC6 in D_2O.
